# Machine learning based prediction of low birth weight and its associated risk factors: Insights from the Bangladesh Demographic and Health Survey 2022

**DOI:** 10.1371/journal.pgph.0005187

**Published:** 2025-09-30

**Authors:** Nourin Sultana, Zeba Afia, Isteaq Kabir Sifat, Shamsuz Zoha, Tajin Ahmed Jisa, Md. Kaderi Kibria

**Affiliations:** 1 Department of Statistics, Hajee Mohammad Danesh Science and Technology University, Dinajpur, Bangladesh; 2 Health Research Group, Department of Statistics, University of Rajshahi, Rajshahi, Bangladesh; Qatar University College of Medicine, QATAR

## Abstract

Low birth weight (LBW) is a major public health concern particularly in low and middle-income countries as it contributes to increased infant mortality and long-term health complications. This study applies and evaluates machine learning (ML) algorithms to predict LBW and identify its key risk factors in Bangladesh. Data were collected from 3,192 complete records of ever-married women aged 15–49 years from the Bangladesh Demographic and Health Survey, 2022. Risk factors for LBW were identified by four feature selection techniques including Boruta-based selection (BFS), LASSO regression, Elastic Net and Random Forest (RF). Six ML algorithms, including Logistic Regression (LR), RF, Decision Tree (DT), Artificial Neural Networks (ANN), Extreme Gradient Boosting (XGB), and Light Gradient Boosting Machine (LGBM) were performed to predict LBW. Model performance was evaluated using accuracy, precision, recall, *F*1-score, AUC, and ROC analysis. SHAP values were utilized to examine the influence of individual features on the model’s prediction. The prevalence of LBW in Bangladesh was 27.8%. Twelve features were identified and the XGB model outperformed the other models by achieving the highest performance in predicting LBW with an accuracy of 80% and area under the curve of 0.761 in holdout (90:10) cross-validation. SHAP analysis revealed that ‘pregnancy duration’ and ‘division’ were the strongest predictors of LBW risk followed by ‘marriage to first birth interval’ ‘ANC visits’ ‘C-section’ and ‘place of delivery’. These findings demonstrate that XGB can serve as an effective tool for predicting LBW and identifying important risk factors that may guide targeted interventions. The insights generated from this study can support public health strategies aimed at reducing LBW prevalence in Bangladesh.

## Introduction

Low birth weight (LBW), defined by the World Health Organization as a birth weight of less than 2,500 grams regardless of gestational age, remains a major global health concern [1,2]. It is a critical indicator of neonatal health and a strong predictor of infant survival, physical growth, cognitive development and long-term susceptibility to chronic illnesses, including cardiovascular disease, obesity, and type 2 diabetes [3–5]. Globally, an estimated 30 million infants which is approximately 23.4% of all live births are born annually with LBW [4], underscoring its significant contribution to neonatal morbidity and mortality.

The burden of LBW is disproportionately high in South Asia, where nearly 28% of newborns are affected [6,7]. Bangladesh is among the most severely impacted countries in this region [7,8]. According to the BDHS, the prevalence of LBW among infants in Bangladesh was approximately 17.7% in 2011 [9], 20% in 2014 [10] and 22.6% in 2017–18 [9], reflecting persistent challenges in maternal and child health [11]. LBW in Bangladesh is predominantly associated with preterm birth, intrauterine growth restriction (IUGR), or both and is influenced by multifactorial determinants including maternal nutrition, limited education, inadequate antenatal care, household poverty, environmental exposures, and regional disparities [12,13]. Reducing the prevalence of LBW is essential for achieving Sustainable Development Goal (SDG) 3, particularly target 3.2, which aims to lower neonatal mortality to 12 per 1,000 live births and under-five mortality to 25 per 1,000 live births by 2030 [14]. As such, the early identification of high-risk groups and factors associated with LBW is vital for effective public health planning and intervention in Bangladesh.

Several epidemiological studies have examined LBW determinants in Bangladesh and other low- and middle-income countries using conventional statistical approaches [4,15–17]. However, such methods often struggle to capture nonlinear relationships and complex interactions among risk factors. Recently, machine learning (ML) techniques have emerged as powerful tools for analyzing multidimensional health data and improving the prediction of adverse outcomes, including LBW [18]. Previously, multiple studies demonstrated the utility of ML in LBW prediction across diverse contexts [9,19–21]. For instance, Reza and Salma (2024) applied various ML classifiers with feature-selection techniques to Bangladesh Demographic and Health Survey (BDHS) 2017–18 data and identified RF with a wrapper method as the top performer [20]. A study in Hamadan, Iran, showed that machine learning classifiers can effectively predict low birth weight, with logistic regression highlighting key maternal and neonatal factors [21]. Ranjib A et al. (2023) XGB ML model can effectively predict LBW with high accuracy offering a valuable tool for early risk identification [19]. In Bangladesh, machine learning approaches, including logistic regression and decision tree classifiers, have been used to predict low birth weight, demonstrating accuracies of up to 87.6% [9]. These findings highlight the promise of ML-based approaches for identifying complex patterns underlying LBW [9,19,21]. Despite these advances, important research gaps remain. Many existing ML-based LBW studies in Bangladesh rely on outdated datasets that may not reflect current socioeconomic and healthcare dynamics, and only a few have incorporated interpretability frameworks to translate predictions into actionable public health insights.

To address these limitations, the present study utilizes the most recent nationally representative data from the BDHS 2022 to apply and evaluate predictive ML models for LBW in Bangladesh. Furthermore, this research incorporates Shapley Additive Explanations (SHAP) to provide interpretability to the ML predictions, enhancing transparency and aiding stakeholders in understanding the contribution of each feature to the outcome. The novelty of this study lies in its integration of up-to-date national data with interpretable ML methods and its inclusion of underexplored macro-level variables such as regional and structural determinants. By identifying the most informative predictors of LBW and providing explainable predictions, this research aims to inform data-driven strategies and targeted interventions for reducing LBW and improving maternal and child health outcomes in Bangladesh.

## Materials and methods

### Data source and study population

This study utilized data extracted from the Bangladesh Demographic and Health Survey (BDHS) conducted in 2022 and is freely accessible online [22]. The 2022 BDHS used the Integrated Multi-Purpose Sampling Master Sample developed by the Bangladesh Bureau of Statistics (BBS) based on the 2011 Population and Housing Census. The sampling frame included enumeration areas (EAs) with information on residence location such as urban city corporation or rural and household estimates along with sketch maps that outlined geographic boundaries. A two-stage stratified sampling design was adopted. In the first stage, 675 EAs were selected with probability proportional to size 237 from urban areas and 438 from rural areas. A complete household listing was then conducted in the selected EAs to create the sampling frame for the second stage. In the second stage, an average of 45 households per EA was systematically selected, resulting in 30,375 households (10,665 urban and 19,710 rural). All eligible women aged 15–49 in the selected households were interviewed using a structured questionnaire covering socio-demographic characteristics, reproductive history, and child health. The BDHS 2022 data are publicly accessible through the Demographic and Health Surveys (DHS) Program and were fully anonymized, thereby exempting the study from additional ethical review.

### Outcome variable

The primary outcome variable in this study is birth weight which is measured in grams. In accordance with the WHO definition, LBW is classified as a birth weight of less than 2500 grams while NBW is defined as 2500 grams or more. For analytical purposes, a binary variable was created, where LBW was coded as 1 and NBW as 0 [22].

### Predictor variables

A diverse set of potential predictors of LBW were considered based on an extensive review of prior literature [9,16,23–36]. The selection of variables was guided by their relevance and frequent citation as risk factors for LBW in similar demographic and epidemiological studies. The considered predictors included the place of residence (urban/rural) [29–31], division (comprised eight administrative division) [9,30,32,33], maternal education (no education to higher) [30–32,34,35], child’s sex (male/female) [24,25,30,34,35], maternal age (15–19 – 45–49 years) [34,36], wealth index (poorest to richest) [24,30–32,35], parity (<5 or ≥5 births) [37–40], contraceptive use history (modern, traditional, non-user intending to use, non-user not intending to use) [36], number of ANC visits (<8 or ≥8) [24,26,31,34], marriage-to-first-birth interval (≤24, 25–48, > 48 months) [23,32,34,36], wanted last child (wanted then, later, or no more) [32], child is twin (Single, 1st of multiple, 2nd of multiple) [5,12,41], child is alive (yes/no) [27,28], C-section (yes/no) [10,42–44], maternal occupations (not working/working) [2,5,40,45], gestational age (7–10 months) [13,46–48], maternal BMI [40,41,49,50], and place of delivery (home, hospital, other) [16,31,34]. A detailed description of each variable, including type (categorical, continuous and discrete), and categorization, is presented in Table 1.

**Table 1:** Predictor names, predictor types, predictor descriptions, along with their categorizations.

Variables names	Variable type	Descriptions	Categorization
Maternal age	Continuous	Maternal age in 5-year groups	(i) 15–19, (ii) 20–24, (iii) 25–29, (iv) 30–34, (v) 35–39, (vi) 40–44, and (vii) 45–49
Division	Nominal	Division	(i) Barisal, (ii) Chittagong, (iii) Dhaka, (iv) Khulna, (v) Mymensingh, (vi) Rajshahi, (vii) Rangpur, and (viii) Sylhet.
Residence	Nominal	Type of place of residence	(i) Urban and (ii) Rural.
Maternal Education	Ordinal	Educational attainment	(i) No education, (ii) Primary, (iii) Secondary, and (iv) Higher
Maternal occupation	Nominal	Maternal occupation	i) Not Working and (ii) Working
Wealth index	Ordinal	Respondents wealth index status	(i) Poorest, (ii) Poorer, (iii) Middle, (iv) Richer, and (v) Richest.
Parity	Nominal	Parity at sterilization	(i)<5 and (ii) ≥5
Child is alive	Nominal	The child is alive	(i) Yes and (ii) No
Gestational age	Discrete	Gestation duration (month)	(i) 7, (ii) 8, (iii) 9 and (iv) 10
Marriage to 1st BI	Nominal	Marriage to 1st birth interval	(i) ≤24, (ii) 25–48, and (iii) >48
Child is twin	Nominal	Single or multiple births	(i)Single, (ii) 1st of multiple, (iii) 2nd of multiple.
Child’s sex	Nominal	Sex of the child	(i) Male and (ii) Female
Number of ANC visit	Nominal	Numbers of antenatal visits	(i) <8, and (ii) ≥8
Maternal BMI	Continuous	Body mass index of mother	--
Wanted last child	Nominal	Wanted child or not	(i)Wanted then, (ii)Wanted later and (iii) Wanted no more
Place of delivery	Nominal	Place of delivery	(i) Home, (ii) Hospital, and (iii) Others
C-section	Nominal	Delivery in cesarean section	(i) Yes and (ii) No
Contraceptive use history	Nominal	Use of contraceptive Method	(i) Modern method, (ii)Traditional, (iii) non user- intend to use and (iv) Not intend to use

### Data preprocessing

#### Data cleaning.

As in most data mining applications, the initial phase of data preprocessing was crucial for ensuring the quality and reliability of the analysis. The original BDHS 2022 dataset consisted of 12,654 live births within five years prior to the survey. For this study, we included only live births within that period for which birth weight information was available and obtained either from health cards or maternal recall. To ensure analytical rigor, records with missing or implausible birth weight values (<500g or>600g) as well as lacking key predictor variables were excluded. Missing values in others categorical variables were imputed using the mode while missing values in continuous variables were imputed using the median value. Outliers in continuous variables were identified and handled using the Interquartile Range (IQR) method. After applying these inclusion and exclusion criteria, along with data refinement procedures, a total of 3192 complete mother-child records were retained for machine learning model development.

#### Feature scaling.

Feature scaling is a critical preprocessing step that ensures numerical variables are on a comparable scale, enhancing model convergence and performance. In this study, min-max normalization was applied to rescale numerical features to a [0,1] range, ensuring that no variable disproportionately influenced the model. This was followed by model exploration, training, performance evaluation, hyper-parameter tuning via cross-validation, optimal model selection, and final deployment for predicting low birth weight outcomes [51,52].

#### Feature selection.

Feature selection is an essential step in machine learning to identify the most relevant predictors for classification tasks [53]. In this study, four techniques were employed: BFS, LASSO regression, Elastic Net, and RF. Boruta is particularly effective in high-dimensional settings, identifying statistically significant variables [54]. LASSO and Elastic Net perform embedded selection by applying regularization during model training, allowing automatic identification and coefficient shrinkage of key features [55,56]. RF constructs multiple decision trees using bootstrapped samples, offers a metric for feature significance based on each feature’s contribution to reducing impurity within the trees [53]. To enhance robustness, the final feature set was derived by integrating the results from all four methods (*r* = 4), ensuring comprehensive evaluation from diverse analytical perspectives.


$$Important\;features={⋃}_{i=\text{1}}^{r}{Identified\;features\;form\;BDHS(\text{2}\text{0}\text{2}\text{2})\;dataset}_{i}$$

#### Data balancing and partition.

The dataset included 3,192 samples, with 27.7% (*n* = 886) classified as LBW and 72.3% (*n* = 2,306) classified as NBW, resulting in class imbalance. To address this, we employed the Adaptive Synthetic Sampling (ADASYN) method, which focuses sample generation on harder-to-classify minority instances, thereby improving classifier sensitivity to rare cases. Compared to the Synthetic Minority Oversampling Technique (SMOTE), which generates synthetic samples evenly across the minority class, ADASYN adaptively targets difficult regions in the feature space, often enhancing detection performance for low-prevalence outcomes [57]. Under-sampling of the majority class was avoided to prevent the loss of valuable LBW instances [58]. Preliminary evaluation indicated that ADASYN achieved higher recall for LBW prediction than SMOTE, while under-sampling led to reduced performance. After applying ADASYN, the final dataset consisted of 4,617 samples (2,311 LBW and 2,306 NBW). To ensure proportional representation of each class, we applied stratified random sampling to split into 70% (*n* = 3232, LBW = 1618) for training and 30% (*n* = 1385, LBW = 693) for testing. The test set was held out entirely from model training and hyper-parameter tuning to provide an unbiased evaluation of final model performance, including AUC-ROC computation.

### Machine learning algorithms

This study employed several well-established machine learning algorithms to predict LBW based on relevant risk factors, including LR, ANN, DT, RF, LGBM, and XGB. These models are capable of capturing complex, non-linear relationships and interactions among predictors that traditional statistical approach may fail to detect (see S1 Appendix for details). The selection of these models was guided by prior literature, where they have consistently demonstrated strong predictive performance in health-related outcomes [9,19]. LR is commonly used for binary classification tasks, providing robust predictions based on risk factors [59]. ANNs replicate the human brain’s structure, enabling decision-making by processing complex patterns through interconnected “neurons” [60]. Decision Trees group similar observations and maximize the distinction between target variables within subsets [61] while RF builds multiple decision trees using bootstrap aggregating (bagging), averaging individual predictions to enhance accuracy and reduce overfitting [62]. LGBM constructs decision trees using gradient-based optimization and combines predictions via weighted averages [63]. Lastly, XGB integrates multiple learning models to improve predictive performance through decision tree ensembles [64]. Compared to traditional statistical models, these ML algorithms offer distinct advantages for LBW prediction because they can handle high-dimensional data, non-linear associations, and interactions among socio-demographic and clinical risk factors, which are common in population health datasets [65].

#### Cross-validation and hyper-parameter tuning.

Within the training set only, we applied grid search combined with repeated 10-fold cross-validation to identify optimal hyper-parameters for each algorithm (see Table 2 for details). In each iteration of cross-validation, the training set was further divided into 10 folds, with 9 folds used for model training and 1-fold for validation. This process was repeated multiple times to ensure robust hyper-parameter selection. The final tuned model was then retrained on the entire training set and evaluated on the independent hold-out test set.

**Table 2:** Hyper parameters tuning of different classifiers using GridSearchCV.

Classifier	Hyper-parameter and value
LR	C = 1.0, penalty = l1, solver = liblinear
DT	max_depth = 15, max_features = ‘sqrt’, ‘log2’criterion = gini, min_samples_split = 20, min_sample_leaf = 1
RF	max_depth = 10, max_features = ‘sqrt’, min_samples_leaf’ = 1, min_samples_split = 10, n_estimators = 100
ANN	model_neurons = 15, model_learning_rate = 0.001, epochs = 20, batch_size = 8
LGBM	learning_rate = 0.1, n_estimators = 20, num_leaves = 31
XGB	colsample_bytree = 0.8, learning_rate = 0.05, max_depth = 3, n_estimators = 100, subsample = 1.0

### Performance evaluation

To evaluate the effectiveness of various machine learning algorithms in our binary classification task, we employed a confusion matrix. This matrix served as the basis for computing several evaluation metrics, including accuracy, precision, recall, *F*1-score, and error rate. These metrics provide valuable insights into the predictive performance of the models and assist in identifying the most suitable one for our application.

Performance metrics equations:


$$Accuracy\;=\frac{\left(Tp+\;TN\right)}{\left(TP+TN+FN+FP\right)}$$

$$Precision\;=\frac{TP}{TP+FP}$$

$$Recall\;or\;Sensitivity\;=\frac{TP}{TP+FN}$$

$$F\text{1}\text{-}Score\;=\;\text{2}\;\frac{Precision\;\times \;Recall}{Precision\;+\;Recall}$$

$$Specificity\;=\frac{TN}{TN+FP}$$

$$Error\;=\;\frac{\left(\;FP+FN\right)}{\left(TP+TN+FN+FP\right)}$$

Where TP (true positives), TN (true negatives), FP (false positives), and FN (false negatives) represent the outcomes of binary classification, two additional evaluation metrics Receiver Operating Characteristic (ROC) and Area Under the Curve (AUC) were also employed to assess the performance of the models. The ROC curve illustrates the balance between the sensitivity (true positive rate) and the fall-out (false positive rate) at various classification thresholds. The AUC, which quantifies the total area under the ROC curve, serves as a summary measure of the model’s discriminative ability. The formula used to compute AUC is given below:


$$AUC=\int_{x=\text{0}}^{\text{1}}\;\text{TPR}\left(FPR^{-\text{1}}(x)\right)dx$$

### Kernel shape model interpretability

The SHapley Additive exPlanations (SHAP) framework is widely considered the benchmark for local interpretability, owing to its solid theoretical foundation and versatile applicability [53]. SHAP was selected over other interpretability techniques, including LIME, Partial Dependence Plots (PDPs), and permutation importance, because it offers both consistency and local accuracy guarantees: the sum of SHAP values across features exactly equals the model’s output for each instance, ensuring faithful attribution of contributions [66,67]. Unlike LIME, which uses local surrogate models and may produce inconsistent explanations due to random sampling and linear approximations. SHAP’s game-theoretic approach provides stable, faithful attribution of feature contributions critical for healthcare prediction tasks requiring precise risk factor interpretation [68]. PDPs capture only average feature effects and overlook interaction effects, while permutation importance may be biased by correlated predictors [66]. Recognized as the benchmark for local interpretability, SHAP combines a solid theoretical foundation with broad applicability. Since its introduction, various adaptations have extended its use across domains while preserving its core assumptions [68]. SHAP values quantify each predictor’s influence, indicating both magnitude and direction (positive or negative) [69]. In our model, positive SHAP values corresponded to LBW predictions, while negative values indicated NBW.

The relative importance of each feature, such as the *k*^th^ risk factor, is calculated using Shapley values, based on the following formula:


$$\begin{array}{c} \emptyset_{k}(v)=\frac{\text{1}}{M!}\sum_{S\subseteq M\{k\}}\;|S|!(M-|S|-\text{1})![v(S\cup \{k\})-v(S)] \end{array}$$

**Fig 1:**
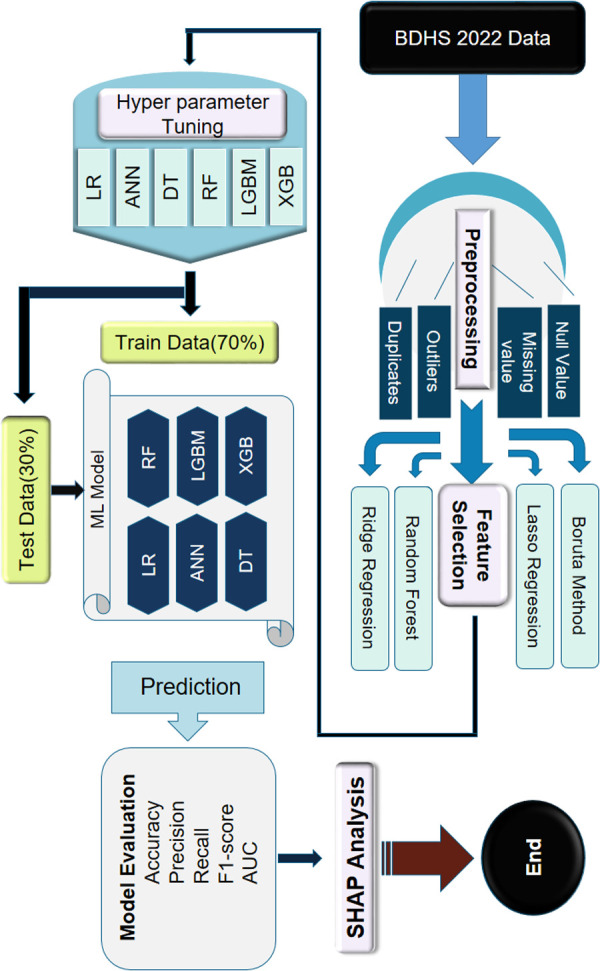
An overview of the comprehensive workflow followed in this study.

Where *S* represents the subset of risk factors that does not include the risk factor for which the value is being calculated; $\emptyset_{k}(v)$, S∪{k} is the subset of risk factors including *k*^th^ risk factor in S, v(S) is the outcome of the ML-based model that explains using the risk factors in *S*; and S⊆M{k} indicates all the sets of *S* that are subset of the entire set of *M* risk factors, excluding *k*^th^ one. The workflow of this study is shown in Fig 1.

### Ethics statement

This study analyzed publicly available data from the Bangladesh Demographic and Health Survey (BDHS) 2022, which is openly accessible online with all identifiable information removed. The survey received ethical approval from the National Research Ethics Committee of Bangladesh. The authors were authorized to use the dataset for independent research purposes. Therefore, no additional ethical approval was required.

## Results

### Characteristics of the respondents:

This study included 3,192 ever-married Bangladeshi women aged 15–49, each with at least one child under five. The overall prevalence of LBW was 27.8% (see Table 3). The mean maternal age was 25.74 years, and the average age at first birth was 19.31 years. About 62.1% resided in rural areas, where the LBW rate was 28%. LBW prevalence declined with increasing maternal education, from 34.7% among uneducated mothers to 25.2% among those with higher education. Economically disadvantaged households showed a higher LBW rate (29.6%), particularly in the poorest wealth. A longer interval (>48 months) between marriage and first birth was linked to higher LBW (30.6%). Fewer antenatal care visits (0–8) were associated with a 28% LBW rate. Home deliveries had a higher LBW prevalence (35.2%) compared to hospital births (27.1%). LBW was more frequent in twin or multiple births (78.9%) and slightly more common in female infants (28.5%) than males (27.1%). Additionally, LBW was higher among non-cesarean deliveries (31%) than C-section (26.2%).

**Table 3:** Characteristics of the study participants (*n* = 3192).

Risk factor	Total, n (%) (n = 3192)	NBW, n(%) (n = 2306; 72.2)	LBW, n(%) (n = 886; 27.8)
Maternal age			
15-19	430(13.5)	310(72.1)	120(27.9)
20-24	1065(33.4)	790(74.2)	275(25.8)
25-29	909(28.5)	661(72.7)	248(27.3)
30-34	545(17.1)	383(70.3)	162(29.7)
35-39	202(6.3)	135(66.8)	67(33.2)
40-44	38(1.2)	25(65.8)	13(34.2)
45-49	3(0.1)	2(66.7)	1(33.3)
Division			
Barisal	328(10.3)	234(71.3)	94(28.7)
Chattogram	490(15.4)	326(66.5)	164(33.5)
Dhaka	527(16.5)	363(68.9)	164(31.1)
Khulna	470(14.7)	370(78.7)	100(21.3)
Mymensingh	338(10.6)	250(74)	88(26)
Rajshahi	363(11.4)	279(76.9)	84(23.1)
Rangpur	389(12.2)	304(78.1)	85(21.9)
Sylhet	287(9)	180(62.7)	107(37.3)
Residence			
Rural	1981(62.1)	1427 (72)	554(28)
Urban	1221(37.9)	879 (72.6)	332 (27.4)
Maternal education			
No education	101(3.2)	66 (65.3)	35(34.7)
Incomplete primary	212(6.6)	146(68.9)	66(31.1)
Complete primary	311(9.7)	216 (69.5)	95 (30.5)
Incomplete secondary	1236(38.7)	906 (73.3)	330 (26.7)
Complete secondary	500(15.7)	350(70)	150(30)
Higher	832 (26.1)	622 (74.8)	210 (25.2)
Maternal occupation			
Yes	754 (23.6)	550 (72.9)	204 (28)
No	2438(76.4)	1756 (72)	682 (46.4)
Wealth index			
Poorest	415 (13)	292 (70.4)	123 (29.6)
Poorer	554(17.4)	389 (70.2)	165 (29.8)
Middle	635 (19.9)	458(72.1)	177 (27.9)
Richer	748 (23.4)	554 (74.1)	194 (25.9)
Richest	840 (26.3)	613 (73)	227 (27)
Parity			
1 birth	1232 (37.5)	634(47.3)	598(52.7)
2 births	1780 (55.2)	1016(57.1)	764 (42.9)
3 and above	234 (7.3)	203(86.8)	31(13.2)
Child is alive			
Yes	3147 (98.6)	2282 (72.5)	865 (27.5)
No	45(1.4)	24 (53.3)	21 (46.7)
Gestational age (months)			
7	19(0.6)	1(5.3)	18(94.7)
8	296(9.3)	166(56.1)	130(43.9)
9	2723(85.3)	2014(74)	709(26)
10	154(4.8)	125((81.2)	29(18.8)
Marriage to Birth interval (months)			
0-24	1748 (54.8)	1251 (71.6)	497 (28.4)
24-48	978(30.6)	730 (74.6)	248 (25.4)
>48	466 (14.6)	325(69.7)	141 (30.6)
Child is twin			
Yes	38 (1.2)	8 (21.1)	30 (78.9)
No	3154 (98.8)	2298(72.9)	856 (27.1)
Child’s sex			
Male	1671 (52.3)	1218 (72.9)	453 (27.1)
Female	1521(47.7)	1088 (71.5)	433(28.5)
Number of ANC visits			
0-8	3082 (96.6)	2220(72)	862 (28)
>8	110(3.4)	86 (78.2)	24 (21.8)
Maternal BMI			
BMI mean ± SD	22.78 ± 4.15	22 ± 3.84	23.70 ± 4.30
Wanted last child			
Wanted then	2633(82.5)	1913(72.7)	720(27.3)
Wanted later	363(11.4)	258(71.1)	105(28.9)
Wanted no more	196(6.1)	135(68.9)	61(31.1)
Place of delivery			
Home	250 (7.8)	162(64.8)	88(35.2)
Hospital	2934(91.9)	2140(72.9)	794(27.1)
Other	8(0.3)	4(50)	4(50)
C-section			
Yes	2137(66.9)	1578(73.8)	559(26.2)
No	1055(33.1)	728(69)	327(31)
Contraceptive use history			
Modern method	2060(64.5)	1500(72.8)	560(27.2)
Traditional method	253(7.9)	177(70)	76(30)
Non user	879(27.53)	629(71.5)	250(28.5)

### Risk factors identification:

Feature selection is a vital step in data preprocessing and a key component of the machine learning pipeline. It involves identifying relevant variables while eliminating irrelevant, redundant, or noisy ones. In this study, four feature selection techniques were utilized to identify significant predictors of LBW. Specifically, BFS identified 12 important features, LASSO identified 14, Elastic Net identified 13, and RF identified 15 (see S1 Table). Based on these results, twelve features were identified as significant risk factors for LBW through the integration of four feature selection techniques (see Table 4 and S1 Table for details). Maternal characteristics included age (15–49 years, grouped into 5-year intervals), educational attainment, and wealth index. Reproductive and clinical factors comprised gestational age (7–10 months), marriage-to-first-birth interval, ANC visits, delivery by C-section, place of delivery, and whether the child was a singleton or part of a multiple birth. Child-level attributes included sex and survival status at birth. Geographic variation was captured by the administrative division of residence. These features collectively represent biological, socio-economic, demographic, and health service–related determinants of LBW risk. These features were then used in model development to enhance predictive accuracy.

**Table 4:** Twelve key risk factors for LBW identified by four feature selection techniques.

SN	Variables	Data type	Descriptions	Categorizations
1.	Maternal age	Ordinal	Maternal age in 5-year groups	(i) 15–19, (ii) 20–24, (iii) 25–29, (iv) 30–34, (v) 35–39, (vi) 40–44, and (vii) 45–49
2.	Division	Nominal	Division	(i) Barisal, (ii) Chittagong, (iii) Dhaka, (iv) Khulna, (v) Mymensingh, (vi) Rajshahi, (vii) Rangpur, and (viii) Sylhet.
3.	Gestational age	Discrete	Gestation time (month)	(i)7, (ii)8, (iii) 9 and (iv) 10
4.	Marriage to 1st BI	Nominal	Marriage to 1st birth interval	(i) ≤24, (ii) 25–48, and (iii) >48
5.	C-section	Nominal	Delivery in cesarean section	(i) Yes and (ii) No
6.	Number of ANC visit	Nominal	Numbers of antenatal visits	(i) <8, and (ii) ≥8
7.	Child is twin	Nominal	Single or multiple births	(i) Single, (ii) 1st of multiple, (iii) 2nd of multiple.
8.	Delivery place	Nominal	Place of delivery	(i) Home, (ii) Hospital, and (iii) Others
9.	Child’s sex	Nominal	Sex of the child	(i) Male and (ii) Female
10.	Maternal Education	Ordinal	Educational attainment	(i) No education, (ii) Primary, (iii) Secondary, and (iv) Higher
11.	Wealth index	Ordinal	Respondents wealth index status	(i) Poorest, (ii) Poorer, (iii) Middle, (iv) Richer, and (v) Richest.
12.	Child is alive	Nominal	The child is alive	(i) Yes and (ii) No

### ML model performance comparison:

**Fig 2:**
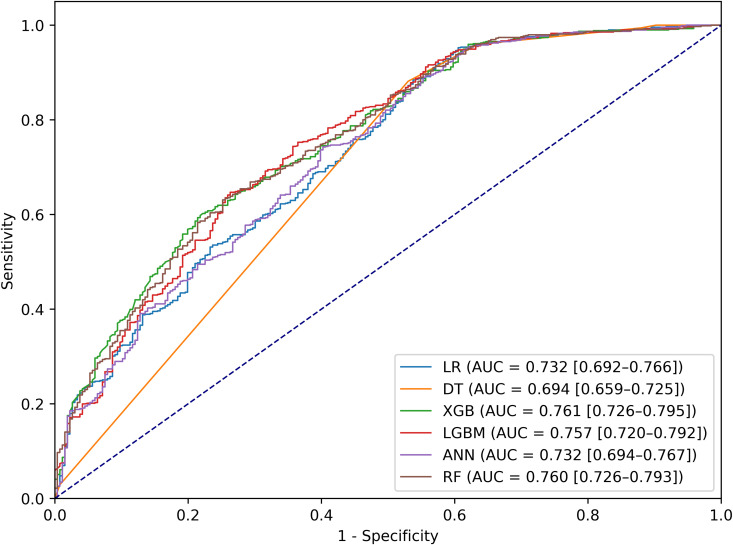
ROC curves of the ML models for predicting LBW using test dataset.

Based on the 12 identified key risk factors, we evaluated ML models and their performance metrics on the training and test datasets, as shown in S2 Table; S1 Fig and Table 5; Fig 2, respectively. Among the models, the DT achieved the highest performance on the training dataset with an accuracy of 83% (see S2 Table for details).

**Table 5:** Comparative performance of machine learning models for predicting low birth weight using the test dataset.

Models	Accuracy	Recall	Precision	F1-Score	AUC
LR	0.78	0.78	0.78	0.77	0.732
RF	0.79	0.78	0.79	0.77	0.760
DT	0.76	0.76	0.74	0.74	0.694
ANN	0.73	0.73	0.70	0.63	0.732
XGB	0.80	0.80	0.79	0.77	0.761
LGBM	0.79	0.79	0.79	0.76	0.757

In the test dataset, XGB demonstrated the best overall performance, with the highest accuracy (0.80), recall (0.80), precision (0.79), *F*1-score (0.77), and AUC (0.761), indicating strong predictive capability. LGBM and RF also performed well, achieving high accuracy (0.79) and F1-scores (0.76 and 0.77, respectively), with AUC values of 0.757 and 0.760, respectively. LR and ANN showed comparable performance, with accuracy ranging from 0.73 to 0.78 and identical AUC values of 0.732. In contrast, DT had the lowest predictive power in the test dataset, with the lowest accuracy (0.76) and AUC (0.694). These findings underscore the superior performance of ensemble-based approaches, particularly XGB, in predicting LBW (see S2 Fig for individual ROC curves of the test dataset).

### Interpretable indicators associated with LBW:

**Fig 3:**
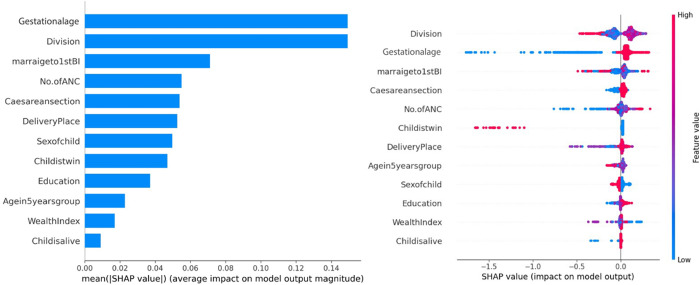
Feature importance of risk factors based on SHAP values, (a) Mean absolute SHAP values (b) Local explanation summary.

A SHAP analysis was performed using the XGB model to identify interpretable socio-demographic and clinical factors associated with LBW. The global feature importance plot (see Fig 3 for details) shows each risk factors overall contribution to the model’s predictions, but it does not distinguish whether a variable increases or decreases the likelihood of LBW. To address this, a SHAP summary plot was used which provides a global-level interpretation of how individual predictors influence LBW. The *x*-axis in the summary plot represents SHAP values, which indicate the magnitude and direction of each variable’s impact on LBW prediction. The SHAP analysis identified multiple significant socio-demographic predictors of LBW. Among them, gestational age’ and ‘division’ showed the strongest association with increased LBW risk. Predictors including ‘marriage-to-first-birth interval’, ‘number of ANC visits’, ‘C-section’, ‘place of delivery’, ‘sex of the child’, ‘twin birth’, and ‘maternal education level’ had moderate contributions. Factors with lower SHAP values, such as ‘maternal age’, ‘wealth index’, and ‘child is alive’, were retained in the model and interpreted in the context of prior evidence. Although their individual contributions were smaller, these variables have known relevance to LBW risk in the literature [4,28,70,71] and including them ensures that subtle but meaningful influences are not overlooked. This approach validates the SHAP outputs and reinforces that both high- and low-contribution features can provide clinically and demographically relevant insights into LBW outcomes in the studied population.

## Discussion

This study provides valuable insights into the key determinants of LBW and evaluates the predictive performance of various ML models based on data from the 2022 Bangladesh Demographic and Health Survey (BDHS) [22]. By leveraging advanced analytical tools, we aimed to identify key socio-demographic and clinical determinants of LBW, while also assessing how ML can be utilized for early prediction and targeted intervention.

The LBW prevalence in our study was 27.8% which is considerably higher than BDHS 2017–18 data (16.3%) [4], as well as in neighboring South Asian countries including India (16.4%) [30,44], Pakistan (16.9%) [48], Nepal (15.3%) [50], and Sri Lanka (14.6%) [40]. This substantial increase in LBW prevalence within Bangladesh highlights a pressing public health concern, warranting further exploration into systemic causes and more effective interventions.

Educational attainment emerged as a compelling determinant of LBW in our study, with mothers lacking formal education being significantly more likely to give birth to LBW infants than those with secondary or higher education mirroring findings from various LMICs where maternal education enhances health literacy, ANC attendance, and nutritional practices [10,16,72]. Supporting this, a study from Ethiopia similarly highlighted the elevated LBW risk among uneducated mothers [41]. In addition, economic status and place of residence were identified as critical predictors, with mothers from poorer households and rural areas exhibiting notably higher LBW rates consistent with findings from Alam et al. [28] and other regional studies [4,28,45]. These disparities likely stem from limited access to quality healthcare services, nutritional support, and skilled birth attendants in underserved communities. Moreover, such inequities may reflect broader structural constraints tied to national economic conditions. In countries with lower GDP per capita, including Bangladesh, healthcare systems are often underfunded, particularly in rural regions, which hamper access to essential maternal health services [73]. Studies have shown that higher GDP per capita is generally linked to lower LBW rates, as greater economic capacity enables improved investment in healthcare infrastructure, maternal education, and social protection programs [42,74]. Hence, macroeconomic development and sustained GDP growth can indirectly contribute to reducing LBW prevalence by enhancing the reach and quality of maternal and child health services.

Interestingly, a longer interval between marriage and first birth was associated with increased LBW risk, a finding that contrasts with the commonly held belief that shorter interpregnancy intervals are more detrimental. However, this result is consistent with previous studies [43,75], which suggest that delayed first births may be associated with latent fertility issues or insufficient maternal preparedness in certain socio-economic contexts. Further qualitative studies are warranted to explore the underlying mechanisms behind this association. Clinically, ANC visits, place of delivery, and delivery type were robust predictors of LBW, with increased ANC visits significantly reducing LBW prevalence likely due to improved maternal-fetal monitoring and timely intervention [47,76]. Home deliveries, conversely, were markedly associated with LBW, reaffirming the protective role of institutional deliveries involving skilled personnel and emergency support [16,49]. Additionally, SHAP analysis further reinforced the importance of gestational age and geographic division as the most influential predictors of LBW [4,46,77]. Shorter gestational durations, a well-established risk factor for LBW [78,79], highlight the critical need for gestational monitoring to prevent preterm births, while the division of residence reflected regional disparities in care access and outcomes suggesting the value of tailored, region-specific health strategies.

In terms of predictive performance, the XGB model outperformed others with 80% accuracy and an AUC of 0.761. Our findings are in concordance with another study conducted in Bangladesh where ensemble models like XGB and RF showed superior performance in healthcare prediction tasks [80]. Comparatively, studies conducted in other LMICs have also reported strong performances using different models. For instance, in the United Arab Emirates, Logistic Regression combined with SMOTE achieved an accuracy of 90.2%, precision of 87.6%, and an F1-score of 0.89 [54]. An Iranian study found that multiple models, including Decision Trees and Neural Networks, attained accuracy levels above 87% for LBW prediction [21], while in Afghanistan, Random Forest achieved the highest performance with an accuracy of 84.7% using their DHS data [81]. Ensemble models like XGB and RF effectively handle high-dimensional data, non-linear associations, and predictor interactions, making them well-suited for LBW prediction. However, a known limitation of such ML models is their lack of interpretability; complex algorithms often function as “black boxes,” making it challenging for clinicians to understand which factors most strongly influence predictions. For example, studies in neonatal and maternal health have highlighted the difficulty in translating ANN or XGB predictions into actionable clinical insights without feature importance analysis or SHAP plots [82,83]. In our study, SHAP analysis was employed to improve interpretability by identifying gestational age and geographic division as the most influential predictors, helping bridge the gap between predictive accuracy and clinical utility. Features with lower SHAP contributions, such as maternal age, wealth index, and child survival status, were also evaluated for relevance based on prior literature [4,28,70,71], ensuring that subtle but meaningful risk factors were not overlooked. This approach validates the SHAP outputs, confirming that both high- and low-contribution features provide clinically and demographically meaningful insights into LBW risk. Another limitation relates to dataset currency. While BDHS 2022 provides nationally representative data, ML models trained on survey datasets may face temporal drift if applied to future populations such as socio-economic conditions, healthcare access, and maternal behaviors change over time. Previous research has shown that predictive models based on older demographic or clinical datasets can perform poorly when applied to more recent populations, underscoring the importance of continual dataset updates and external validation [84].

Internal validity was reinforced through several methodological safeguards. The analysis used a nationally representative BDHS 2022 dataset, minimizing measurement bias. Preprocessing steps included mode and median imputation for missing values, IQR-based outlier handling, and class balancing with ADASYN enhanced data quality. Stratified random sampling preserved class proportions in training and testing subsets, while repeated 10-fold cross-validation limited overfitting. Importantly, all performance metrics, including AUC-ROC, were computed on an independent hold-out test set, providing an unbiased assessment of model performance. External validity is supported by the dataset’s coverage of urban and rural populations across Bangladesh, enhancing national generalizability. However, performance may differ in other populations or future BDHS waves due to demographic, socio-economic, and healthcare variations. Cultural and regional differences in maternal health behaviors may also influence predictive relationships. Therefore, external validation using independent datasets from different contexts is recommended to confirm robustness and applicability.

### Limitations

While this study offers valuable insights, several limitations must be considered. First, the cross-sectional nature of the data restricts the ability to infer causal relationships between the identified factors and LBW. Additionally, the use of self-reported information provided by mothers without independent verification from healthcare professionals introduces the possibility of recall bias. Furthermore, key variables found to be significant in other studies, such as maternal comorbidities, were not captured in the BDHS-2022 dataset, limiting the scope of analysis. Decision Tree’s training accuracy (83%) and test accuracy (76%) suggests overfitting. This is a known issue for single, unregularized trees, which tend to memorize training data. To reduce this risk, we applied constraints such as limiting maximum depth and setting minimum sample requirements for node splits. To address these gaps, future research should integrate clinical evaluations conducted by medical professionals to achieve a more robust and comprehensive understanding of the determinants of LBW in Bangladesh.

## Conclusions

This study applied advanced machine learning techniques to nationally representative BDHS 2022 data to identify key predictors of LBW. Maternal education, household economic status, rural residence, antenatal care utilization, delivery conditions, and gestational duration emerged as the most influential determinants, underscoring persistent socio-economic and geographic disparities in maternal health. The XGB model demonstrated the highest predictive performance, highlighting the potential of machine learning for identifying at-risk populations and informing targeted interventions. These findings emphasize the need for public health strategies that prioritize maternal education, equitable access to antenatal and institutional delivery services, and region-specific approaches. Strengthening healthcare delivery systems alongside broader economic investments remains crucial to reducing LBW and improving maternal and neonatal health outcomes in Bangladesh and comparable LMIC settings.

## Supporting information

S1 Fig ROC curves of the ML models for predicting LBW using the training dataset. (TIFF)

S2 Fig ROC curves of ML model for predicting LBW in the test dataset: (a) Logistic Regression, (b) Random Forest, (c) Decision Tree, (d) Artificial Neural Network, (e) Extreme Gradient Boosting and (f) Light Gradient Boosting Machine. (TIF)

S1 Table Features identified by four feature selection techniques. (DOCX)

S2 Table Comparative performance of machine learning models for predicting low birth weight using the training dataset. (DOCX)

S1 Appendix Detailed description of machine learning algorithms. (DOCX)

S1 Code Code used for data analysis in this study. (TXT)

S1 Data Data used for the analyses in this study. (SAV)
