# Supplementary figures and images for: Machine learning based prediction of low birth weight and its associated risk factors: Insights from the Bangladesh Demographic and Health Survey 2022
Source: PLOS Glob Public Health. 2025 Sep 30;5(9):e0005187. doi: 10.1371/journal.pgph.0005187 (PMC12483264; doi:10.1371/journal.pgph.0005187)

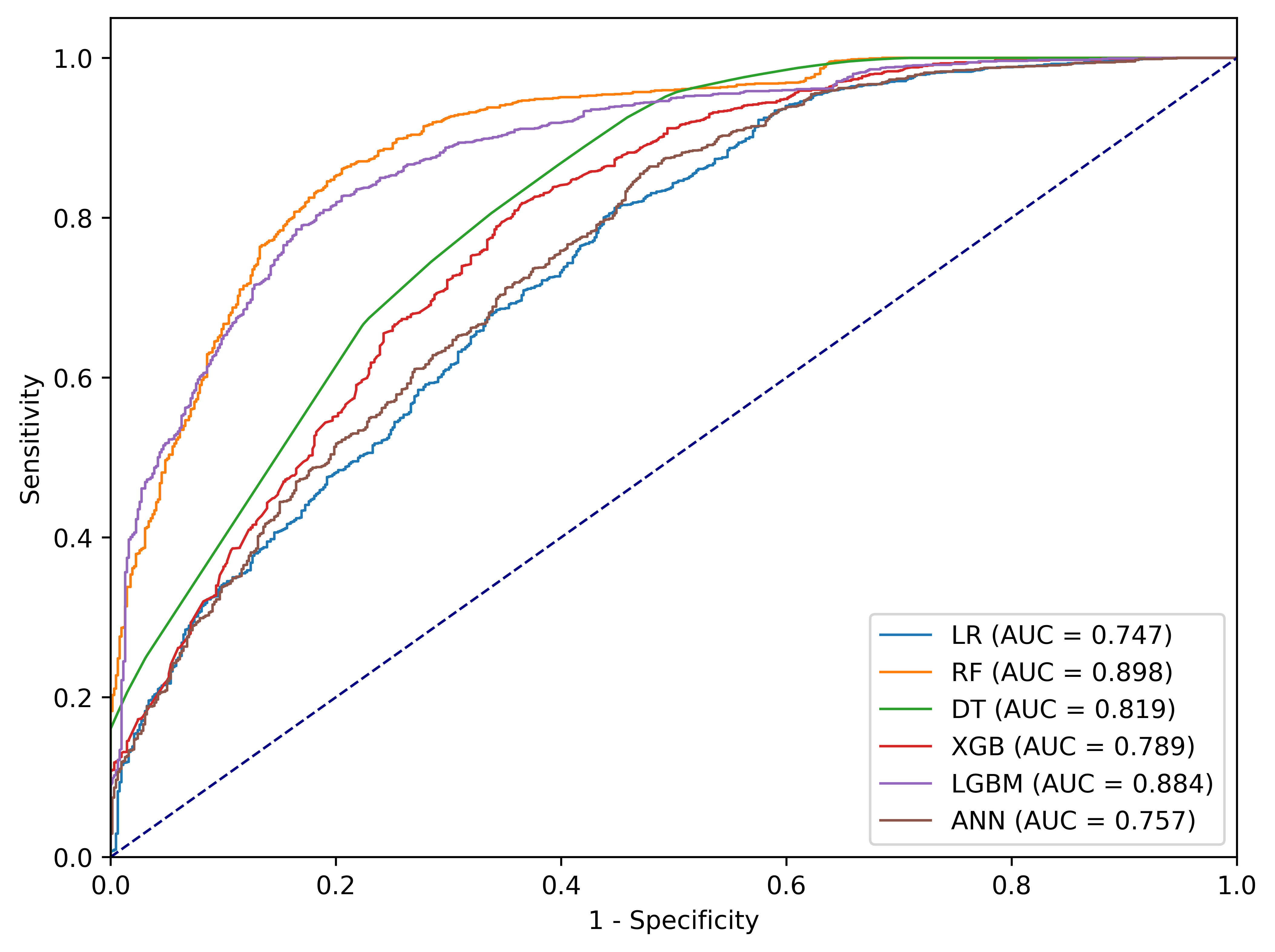

Supplement: S1 Fig — (TIFF) [file pgph.0005187.s001.tiff]

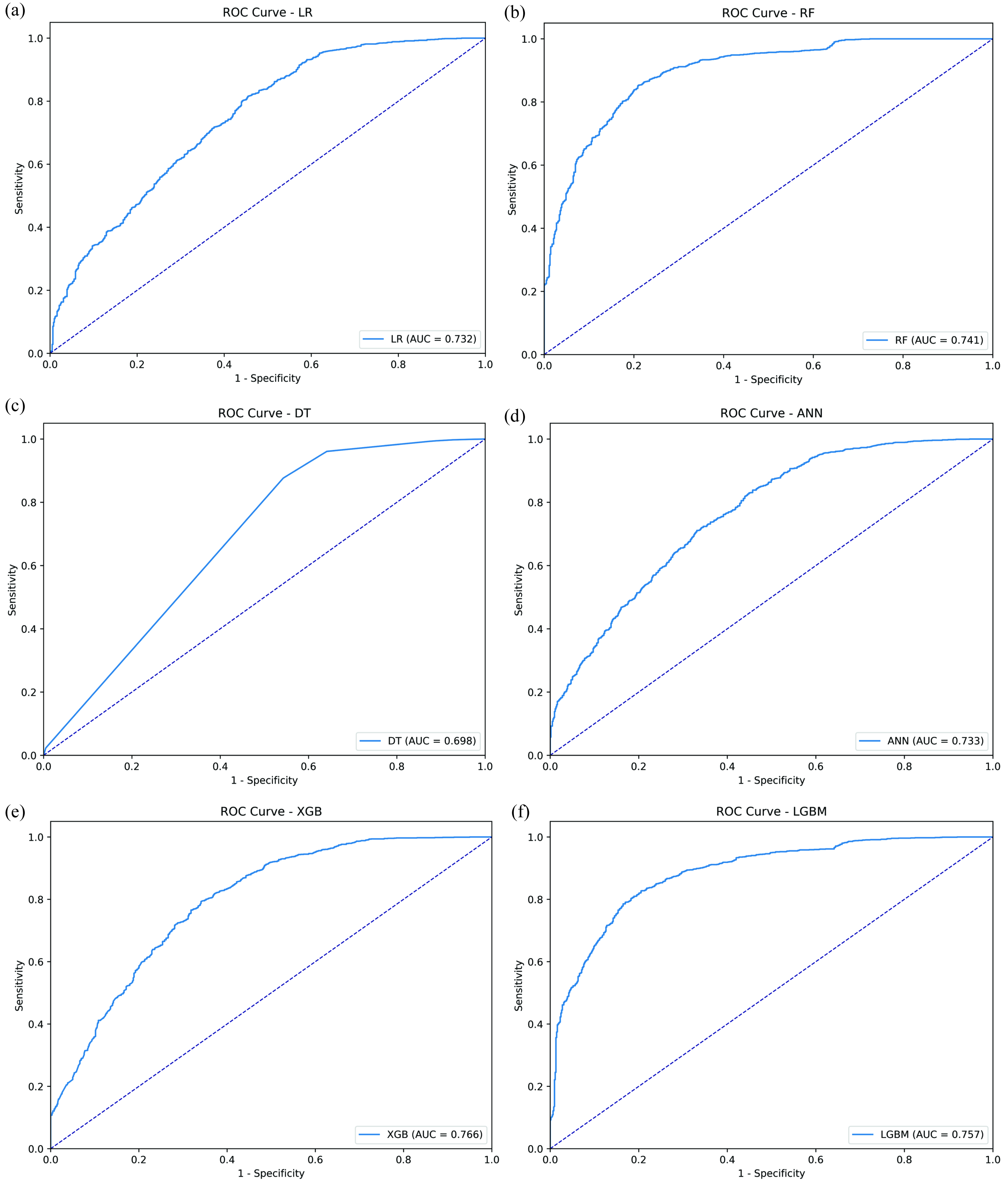

Supplement: S2 Fig — (TIF) [file pgph.0005187.s002.tif]
