# Supplementary material for: Machine learning based prediction of low birth weight and its associated risk factors: Insights from the Bangladesh Demographic and Health Survey 2022
Source: PLOS Glob Public Health. 2025 Sep 30;5(9):e0005187. doi: 10.1371/journal.pgph.0005187 (PMC12483264; doi:10.1371/journal.pgph.0005187)
Supplement: S1 Table — (DOCX) [file pgph.0005187.s003.docx]

**S1 Table:** Feature identified by four feature selection techniques

| **Feature selection technique** | **Selected Features** |
| --- | --- |
| BFS approach | Mother’s age, Division, Gestational age, Marriage to 1st BI, Delivery by CS, ANC visit, Child is twin, Delivery place, Child’s Sex, Education, Wealth index and Child is alive. |
| LASSO regresssion | Mother’s age, Gestational age, Marriage to 1st BI, Delivery by CS, ANC visit, Child is twin, Delivery place, Child’s Sex, Education, Wealth index and Child is alive. |
| Elastic Net | Mother’s age, Division, Gestational age, Marriage to 1st BI, Delivery by CS, ANC visit, Child is twin, Child’s Sex, Education, and Child is alive. |
| Random Forest | Mother’s age, Division, Gestational age, Marriage to 1st BI, ANC visit, Child is twin, Child’s Sex, Wealth index and Child is alive. |
| Selected Features | Mother’s age, Division, Gestational age, Marriage to 1st BI, Delivery by CS, ANC visit, Child is twin, Delivery place, Child’s Sex, Education, Wealth index and Child is alive. |
