# Supplementary material for: Machine learning based prediction of low birth weight and its associated risk factors: Insights from the Bangladesh Demographic and Health Survey 2022
Source: PLOS Glob Public Health. 2025 Sep 30;5(9):e0005187. doi: 10.1371/journal.pgph.0005187 (PMC12483264; doi:10.1371/journal.pgph.0005187)
Supplement: S2 Table — (DOCX) [file pgph.0005187.s004.docx]

**S2 Table:** Comparative performance of machine learning models in predicting low birth weight using the training dataset

| **Models** | **Accuracy** | **Recall** | **Precision** | **F1-Score** | **AUC** |
| --- | --- | --- | --- | --- | --- |
| LR | 0.79 | 0.79 | 0.81 | 0.78 | 0.747 |
| ANN | 0.74 | 0.74 | 0.76 | 0.65 | 0.757 |
| DT | 0.83 | 0.83 | 0.83 | 0.82 | 0.819 |
| RF | 0.81 | 0.81 | 0.79 | 0.77 | 0.898 |
| XGB | 0.80 | 0.80 | 0.80 | 0.77 | 0.789 |
| LGBM | 0.80 | 0.80 | 0.79 | 0.77 | 0.884 |
