# Supplementary material for: Machine learning based prediction of low birth weight and its associated risk factors: Insights from the Bangladesh Demographic and Health Survey 2022
Source: PLOS Glob Public Health. 2025 Sep 30;5(9):e0005187. doi: 10.1371/journal.pgph.0005187 (PMC12483264; doi:10.1371/journal.pgph.0005187)
Supplement: S1 Appendix — (DOCX) [file pgph.0005187.s005.docx]

**Machine Learning Algorithms**

**Logistic Regression (LR)**

LR is a supervised learning method and a statistical model used to predict the probability of a binary outcome, where the target variable has two categories: *success* (1) and *non-success* (0) [1]. This model utilizes the logistic function to predict the likelihood of the response (HTN and non-HTN) based on various input features. The logistic function can be represented as follows

$$Logit\left( p_{j} \right)=log\left( \frac{p_{j}}{1-p_{j}} \right)=\beta_{0}+\beta_{1}x_{1j}+\beta_{2}x_{2j}+...+\beta_{k}x_{kj}+\epsilon_{j}, j=1,2,...,n\text{ }$$

Where,$p_{j}$denote the probability of HTN for *j^th^* individual;$X_{kj}$is the *k^th^* input feature of the *j^th^* individual and$\beta_{k}$is the *k^th^* regression coefficients.

The above equation can be expressed as

$$P=\frac{exp\left( \beta_{0}+\beta_{1}+\beta_{2}+...+\beta_{k}x_{kj} \right)}{1+exp\left( \beta_{0}+\beta_{1}+\beta_{2}+...+\beta_{k}x_{kj} \right)}\text{ }$$

and odds as

$$\frac{p}{1-p}=exp\left( \beta_{0}+\beta_{1}+\beta_{2}+...+\beta_{k}x_{kj} \right)\text{ }$$

If $\frac{p}{1-p}>1,$then we classify as HTN, while $\frac{p}{1-p}<1,$then we classify as non-HTN.

**Decision Tree (DT)**

DT is a supervised learning algorithm that predicts outcomes by recursively splitting the dataset into subsets based on feature values. The model is structured as a tree, where internal nodes represent decision rules, branches represent possible outcomes of those rules, and leaf nodes correspond to final predictions or class labels.

DTs aim to create partitions that maximize the homogeneity of target variables within each node. This is often achieved using splitting criteria such as Gini impurity or information gain.

Once trained, DTs are easy to interpret, as the prediction process follows a simple path from the root node to a leaf. However, they can be prone to overfitting if the tree grows too deep without constraints such as maximum depth, minimum samples per split, or pruning strategies.

Mathematically, a decision tree seeks to find the feature *X_j_* and split point *S* that minimize an

impurity function *Q* across child nodes:

$$\min_{j,s} \left[ \frac{n_{left}}{n}Q_{left}\left( s \right)+ \frac{n_{right}}{n}Q_{right}\left( s \right) \right]$$

Where $n_{left}$​ and $n_{right}$ are the number of samples in the left and right child nodes after the split, and n is the total number of samples at the current node.

**Artificial Neural Network (ANN)**

ANN is a mathematical model inspired by the functional characteristics of biological neural networks [2]. It consists of interconnected processing nodes (neurons) organized into three types of layers: input, hidden, and output. The input layer connects to the hidden layer, and the hidden layer connects to the output layer through weighted links. These weights are updated during training to improve predictive accuracy.

Training is commonly performed using the backpropagation (BP) algorithm, a gradient descent–based method that minimizes the total error by adjusting the weights. Training stops when the mean square error (MSE) ceases to decrease and starts to increase, which signals overfitting [3].

The MSE is calculated as:

$$MSE=1/n\sum_{i=1}^{n} \left( d_{i}-O_{i} \right)^{2}$$

where *n* is the number of output data points, *O*_i_​ is the network’s output value, and did *d*_i_ is the target (true) value.

**Random Forest (RF)**

RF is an ensemble machine learning method that uses multiple decision trees as base classifiers [4]. Proposed by Breiman, RF builds a collection of tree-based predictors, where each tree is trained on a dataset generated from a random vector 𝛩𝑘 ​, sampled independently but from the same distribution as the other trees.

In this framework, the *k*th tree is represented as ℎ(𝑋, 𝛩_𝑘_), where *X* is the input vector. The predictions of all trees are averaged to produce the final output, which increases predictive accuracy and reduces overfitting:

$$\hat{Y}=1/n\sum_{k=1}^{n} h_{k}\left( X \right)$$

when 1 ≤ 𝑘 < 𝑛, 𝑛 is the total number of trees formed, and $\hat{Y}$ is the predicted target value.

The mean-squared generalization error for any tree ℎ(X) is:

$$E_{X,Y}\left( Y-h\left( X \right) \right)^{2}$$

For an infinite forest, the average prediction converges as:

$$E_{X,Y}\left( Y-{av}_{k}h\left( X,\Theta_{k} \right) \right)^{2}\to E_{X},Y\left( Y-E_{\theta}h\left( X,\Theta\right) \right)^{2}$$

Where ${av}_{k}h\left( X,\Theta_{k} \right)$ is the average prediction over all trees in the forest.

**Extreme Gradient Boosting (XGB)**

XGB is a powerful ensemble-based machine learning algorithm belonging to the family of boosting methods. Boosting aims to build a strong classifier by sequentially combining multiple weak learners (typically decision trees), where each subsequent learner focuses on correcting the errors of its predecessors [5,6].

In XGB, the original dataset is partitioned into several subsets, and each base learner (weak classifier) is trained on these subsets. The outputs of these learners are weighted and summed to produce the final prediction. Through iterative training, later classifiers adjust for the misclassifications made by earlier ones, improving overall accuracy. This process continues until the model adequately predicts the target variable’s class membership.

Mathematically, the model’s prediction is expressed as:

$$\hat{y}=\phi\left( x \right)=\frac{1}{n}\sum_{k=1}^{n} f_{k}\left( x \right)$$

where $\hat{y}$ is the predicted value. $f_{k}\left( x \right)$ represents the *kth* function (tree) learned by the model, and nnn is the total number of trees.

The set of functions $f_{k}\left( x \right)$ is learned by minimizing the regularized objective function:

$$L\left( \phi\right)=\sum_{i} l\left( \hat{y_{i}},y_{i} \right)+\sum_{k} \Omega\left( f_{K} \right)$$

where $l\left( \hat{y_{i}},y_{i} \right)$ is the loss function measuring the difference between the predicted and actual values, and $\Omega\left( f_{K} \right)$ is a regularization term that controls model complexity, reducing overfitting [7].

**Light Gradient Boosting Machine (LGBM)**

The advanced LightGBM (LGBM) algorithm has been applied to predict hypertension. LGBM is a gradient boosting framework that builds tree-based models in an additive fashion, where each new tree predicts the residuals of previous trees. It is optimized for efficiency and scalability using Gradient-based One-Side Sampling (GOSS) and Exclusive Feature Bundling (EFB) [8]. Unlike XGBoost, LGBM employs histogram-based algorithms to discretize continuous features into bins, reducing memory usage and speeding up training, and follows a leaf-wise growth strategy with depth constraints [9]. Its main difference from the XGBoost model is that it uses histogram-based algorithms to speed up the training process, reduce memory consumption and employ a leaf-wise growth strategy with depth constraints. The basic idea of histogram algorithm is to discretize continuous floating-point eigenvalues into 𝑘 bins and construct a histogram with a width of 𝑘 [10]. LGBM algorithm is based on decision tress therefore the formulation of the model is as follows. Given a training data set $S=\left( x_{i},y_{i} \right);i=1,2,\cdot\cdot\cdot,n;x_{i}\in R^{m},y_{i}\in R$ where *n* is the sample with *m* features. To find the estimation, the decision tress predictions are combined as follows;

$$\hat{y}_{i}^{LG}=\sum_{p=1}^{p} f_{p}\left( xi \right)$$

where the number of trees is *p* with *f_p_* as trees. The goal is to minimize the objective function below to obtain *f_p_.*

$$f_{p}=arg\min_{f_{p}}\sum_{i=1}^{1} L\left( y_{i},{ŷ}_{i}^{LG\left( p \right)} \right)+\Omega\left( f_{p} \right)$$

The loss function is *L* and the regularization parameter **Ω** which is given by

$$\Omega\left( f_{p} \right)=\alpha T+\frac{1}{2}\lambda\sum_{j=1}^{T} w_{j}^{2}$$

Where *α* and *λ* are the penalty parameters for *T* leaves and weight of leaves *w*. Taking *L* as a loss function which is a squared error, then

${L\left( y_{i},\hat{y}_{i}^{LG\left( p-1 \right)}+f_{p}\left( x \right) \right)=\left( y_{i}-\hat{y}_{i}^{LG\left( p-1 \right)}-f_{p}\left( x \right) \right)}^{2}\left( r-f_{p}\left( x \right) \right)^{2}$

the residual *r* is fitted to obtain *f_p_*. The function for minimizing the objective function at iteration *p* is defined using a quadratic approximation as

$f_{p}\simeq arg{\min_{f}}_{p}\sum_{i=1}^{n} \left[ g_{i} | f_{p}\left( x_{i} \right)+1/2h_{i}f_{i}^{2}\left( x_{i} \right) \right]+\Omega\left( f_{p} \right)$,

$g_{i}=\partial_{\hat{y}_{i}^{LG\left( p-1 \right)}}L\left( y_{i},{ŷ}_{i}^{LG\left( p-1 \right)} \right)$ ,

$h_{i}=\partial_{\hat{y}_{i}^{LG\left( p-1 \right)}}^{2}L\left( y_{i},{ŷ}_{i}^{LG\left( p-1 \right)} \right)$,

Through minimizing the objective function, a new tree *f_p_* is obtained. Each mode with the biggest information gain is divided by the decision tree. The variance gain for a node that separates feature *j* at point s is given by

$$Z_{j\mid0}(s)=\frac{1}{n_{0}}\left\{ \frac{\left( \sum_{\left\{ x\in\in O:x_{i}\leq s \right\}} g_{i} \right)^{2}}{n_{l|0|}^{j}(s)}+\frac{\left( \sum_{\left\{ x_{i}\in O:x_{j}>s \right.} g_{i} \right)^{2}}{n_{r|0|}^{j}(s)} \right\},$$

O is samples on the decision tree fixed node $n_{o}=\sum I\left[ x_{i}\in O \right]$,

$n_{l/o}^{j}\left( s \right)=\sum I\left[ x_{i}\in O:x_{ij}\leq s \right]$ and $n_{l/o}^{j}\left( s \right)=\sum I\left[ x_{i}\in O:x_{ij}\leq s \right]$. The decision tree selects$s_{j}*arg\max_{s}z_{j}\left( s \right)$ for each feature *j* and computes the highest gain Z_i_(s_j_^*^). The data will be split into the right and left nodes according to feature *j** at point *s_j_^*^*. All samples are scanned to find the optimal splitting point in order to calculate the information gain.

References

1. Priya Ranganathan; C. S. Pramesh; Rakesh Aggarwal Common Pitfalls in Statistical Analysis: Logistic Regression. *Perspect. Clin. Res.* **2017**, 148–151.

2. *The Handbook of Brain Theory and Neural Networks*; 2003; Vol. 41;.

3. Montesinos López, O.A.; Montesinos López, A.; Crossa, J. Fundamentals of Artificial Neural Networks and Deep Learning BT - Multivariate Statistical Machine Learning Methods for Genomic Prediction. **2022**, 379–425.

4. Andy, L.; Matthew, W. Classification and Regression by RandomForest. *R News* **2002**, *2*, 18–22.

5. Chen, T.; Guestrin, C. XGBoost: A Scalable Tree Boosting System. *Proc. ACM SIGKDD Int. Conf. Knowl. Discov. Data Min.* **2016**, *13*-*17*-*Augu*, 785–794, doi:10.1145/2939672.2939785.

6. Islam, M.M.; Alam, M.J.; Maniruzzaman, M.; Ahmed, N.A.M.F.; Ali, M.S.; Rahman, M.J.; Roy, D.C. Predicting the Risk of Hypertension Using Machine Learning Algorithms: A Cross Sectional Study in Ethiopia. *PLoS One* **2023**, *18*, doi:10.1371/journal.pone.0289613.

7. C. CHEN, T.. G. XGBoost: A Scalable Tree Boosting System. Proceedings of the 22nd ACM SIGKDD International Conference on Knowledge Discovery and Data Mining. *San Fr. Calif.* **2016**.

8. Ke, G.; Meng, Q.; Finley, T.; Wang, T.; Chen, W.; Ma, W.; Ye, Q.; Liu, T.Y. LightGBM: A Highly Efficient Gradient Boosting Decision Tree. *Adv. Neural Inf. Process. Syst.* **2017**, *2017*-*Decem*, 3147–3155.

9. Chen, T.; Xu, J.; Ying, H.; Chen, X.; Feng, R.; Fang, X.; Gao, H.; Wu, J. Prediction of Extubation Failure for Intensive Care Unit Patients Using Light Gradient Boosting Machine. *IEEE Access* **2019**, *7*, 150960–150968, doi:10.1109/ACCESS.2019.2946980.

10. Chakraborty, D.; Elhegazy, H.; Elzarka, H.; Gutierrez, L. A Novel Construction Cost Prediction Model Using Hybrid Natural and Light Gradient Boosting. *Adv. Eng. Informatics* **2020**, *46*, doi:10.1016/j.aei.2020.101201.
